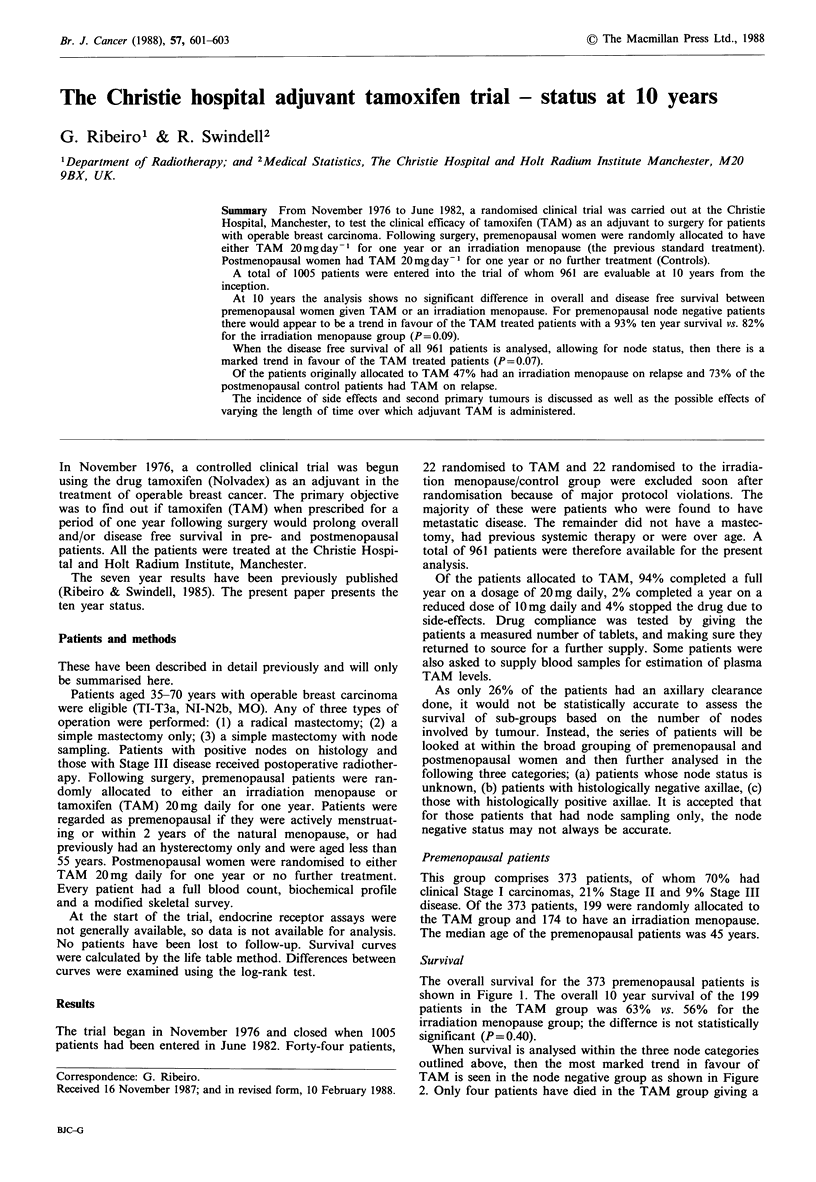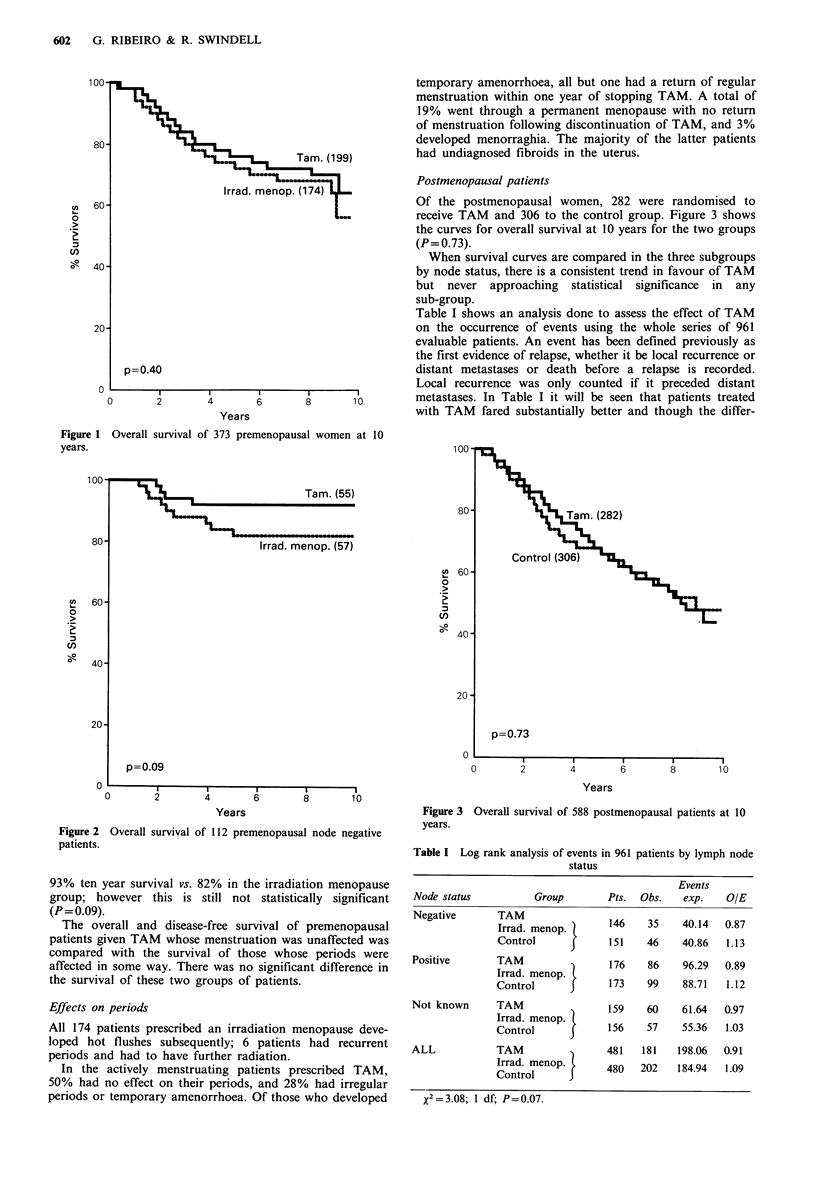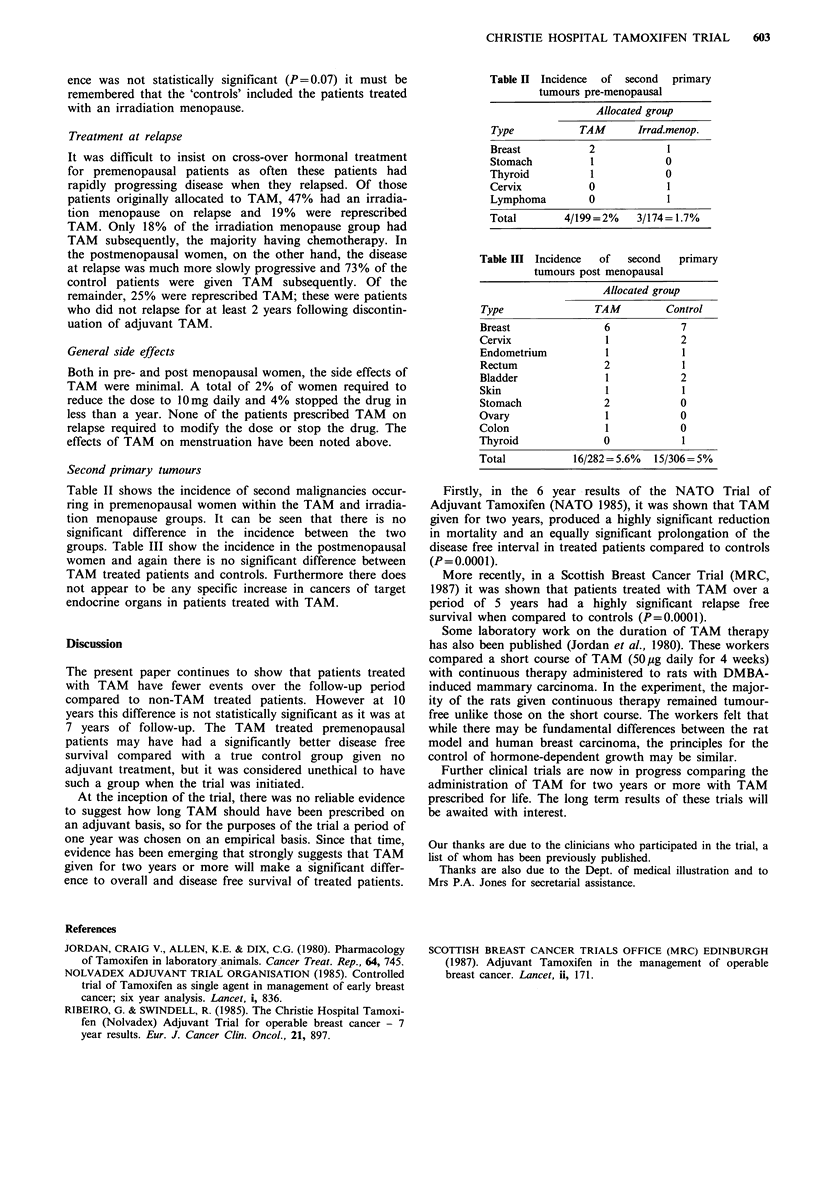# The Christie hospital adjuvant tamoxifen trial--status at 10 years.

**DOI:** 10.1038/bjc.1988.136

**Published:** 1988-06

**Authors:** G. Ribeiro, R. Swindell

**Affiliations:** Department of Radiotherapy, Christie Hospital, Manchester, UK.

## Abstract

From November 1976 to June 1982, a randomised clinical trial was carried out at the Christie Hospital, Manchester, to test the clinical efficacy of tamoxifen (TAM) as an adjuvant to surgery for patients with operable breast carcinoma. Following surgery, premenopausal women were randomly allocated to have either TAM 20 mg day-1 for one year or an irradiation menopause (the previous standard treatment). Postmenopausal women had TAM 20 mg day-1 for one year or no further treatment (Controls). A total of 1005 patients were entered into the trial of whom 961 are evaluable at 10 years from the inception. At 10 years the analysis shows no significant difference in overall and disease free survival between premenopausal women given TAM or an irradiation menopause. For premenopausal node negative patients there would appear to be a trend in favour of the TAM treated patients with a 93% ten year survival vs. 82% for the irradiation menopause group (P = 0.09). When the disease free survival of all 961 patients is analysed, allowing for node status, then there is a marked trend in favour of the TAM treated patients (P = 0.07). Of the patients originally allocated to TAM 47% had an irradiation menopause on relapse and 73% of the postmenopausal control patients had TAM on relapse. The incidence of side effects and second primary tumours is discussed as well as the possible effects of varying the length of time over which adjuvant TAM is administered.


Br. J. Cancer (1988), 57, 601–603

© The Macmillan Press Ltd., 1988

### The Christie hospital adjuvant tamoxifen trial – status at 10 years

G. Ribeiro[1] & R. Swindell[2]

[1]*Department of Radiotherapy; and* [2]*Medical Statistics, The Christie Hospital and Holt Radium Institute Manchester, M20 9BX, UK.*

**Summary** From November 1976 to June 1982, a randomised clinical trial was carried out at the Christie Hospital, Manchester, to test the clinical efficacy of tamoxifen (TAM) as an adjuvant to surgery for patients with operable breast carcinoma. Following surgery, premenopausal women were randomly allocated to have either TAM 20 mg $day^{-1}$ for one year or an irradiation menopause (the previous standard treatment). Postmenopausal women had TAM 20 mg $day^{-1}$ for one year or no further treatment (Controls).

A total of 1005 patients were entered into the trial of whom 961 are evaluable at 10 years from the inception.

At 10 years the analysis shows no significant difference in overall and disease free survival between premenopausal women given TAM or an irradiation menopause. For premenopausal node negative patients there would appear to be a trend in favour of the TAM treated patients with a 93% ten year survival *vs.* 82% for the irradiation menopause group ($P=0.09$).

When the disease free survival of all 961 patients is analysed, allowing for node status, then there is a marked trend in favour of the TAM treated patients ($P=0.07$).

Of the patients originally allocated to TAM 47% had an irradiation menopause on relapse and 73% of the postmenopausal control patients had TAM on relapse.

The incidence of side effects and second primary tumours is discussed as well as the possible effects of varying the length of time over which adjuvant TAM is administered.

In November 1976, a controlled clinical trial was begun using the drug tamoxifen (Nolvadex) as an adjuvant in the treatment of operable breast cancer. The primary objective was to find out if tamoxifen (TAM) when prescribed for a period of one year following surgery would prolong overall and/or disease free survival in pre- and postmenopausal patients. All the patients were treated at the Christie Hospital and Holt Radium Institute, Manchester.

The seven year results have been previously published (Ribeiro & Swindell, 1985). The present paper presents the ten year status.

### Patients and methods

These have been described in detail previously and will only be summarised here.

Patients aged 35–70 years with operable breast carcinoma were eligible (TI-T3a, NI-N2b, MO). Any of three types of operation were performed: (1) a radical mastectomy; (2) a simple mastectomy only; (3) a simple mastectomy with node sampling. Patients with positive nodes on histology and those with Stage III disease received postoperative radiotherapy. Following surgery, premenopausal patients were randomly allocated to either an irradiation menopause or tamoxifen (TAM) 20 mg daily for one year. Patients were regarded as premenopausal if they were actively menstruating or within 2 years of the natural menopause, or had previously had an hysterectomy only and were aged less than 55 years. Postmenopausal women were randomised to either TAM 20 mg daily for one year or no further treatment. Every patient had a full blood count, biochemical profile and a modified skeletal survey.

At the start of the trial, endocrine receptor assays were not generally available, so data is not available for analysis. No patients have been lost to follow-up. Survival curves were calculated by the life table method. Differences between curves were examined using the log-rank test.

### Results

The trial began in November 1976 and closed when 1005 patients had been entered in June 1982. Forty-four patients, 22 randomised to TAM and 22 randomised to the irradiation menopause/control group were excluded soon after randomisation because of major protocol violations. The majority of these were patients who were found to have metastatic disease. The remainder did not have a mastectomy, had previous systemic therapy or were over age. A total of 961 patients were therefore available for the present analysis.

Of the patients allocated to TAM, 94% completed a full year on a dosage of 20 mg daily, 2% completed a year on a reduced dose of 10 mg daily and 4% stopped the drug due to side-effects. Drug compliance was tested by giving the patients a measured number of tablets, and making sure they returned to source for a further supply. Some patients were also asked to supply blood samples for estimation of plasma TAM levels.

As only 26% of the patients had an axillary clearance done, it would not be statistically accurate to assess the survival of sub-groups based on the number of nodes involved by tumour. Instead, the series of patients will be looked at within the broad grouping of premenopausal and postmenopausal women and then further analysed in the following three categories; (a) patients whose node status is unknown, (b) patients with histologically negative axillae, (c) those with histologically positive axillae. It is accepted that for those patients that had node sampling only, the node negative status may not always be accurate.

### *Premenopausal patients*

This group comprises 373 patients, of whom 70% had clinical Stage I carcinomas, 21% Stage II and 9% Stage III disease. Of the 373 patients, 199 were randomly allocated to the TAM group and 174 to have an irradiation menopause. The median age of the premenopausal patients was 45 years.

### *Survival*

The overall survival for the 373 premenopausal patients is shown in Figure 1. The overall 10 year survival of the 199 patients in the TAM group was 63% *vs.* 56% for the irradiation menopause group; the differnce is not statistically significant ($P=0.40$).

When survival is analysed within the three node categories outlined above, then the most marked trend in favour of TAM is seen in the node negative group as shown in Figure 2. Only four patients have died in the TAM group giving a

Correspondence: G. Ribeiro.

Received 16 November 1987; and in revised form, 10 February 1988.

**Figure 1** Overall survival of 373 premenopausal women at 10 years.

**Figure 2** Overall survival of 112 premenopausal node negative patients.

93% ten year survival *vs.* 82% in the irradiation menopause group; however this is still not statistically significant ($P$=0.09).

The overall and disease-free survival of premenopausal patients given TAM whose menstruation was unaffected was compared with the survival of those whose periods were affected in some way. There was no significant difference in the survival of these two groups of patients.

*Effects on periods*

All 174 patients prescribed an irradiation menopause developed hot flushes subsequently; 6 patients had recurrent periods and had to have further radiation.

In the actively menstruating patients prescribed TAM, 50% had no effect on their periods, and 28% had irregular periods or temporary amenorrhoea. Of those who developed temporary amenorrhoea, all but one had a return of regular menstruation within one year of stopping TAM. A total of 19% went through a permanent menopause with no return of menstruation following discontinuation of TAM, and 3% developed menorraghia. The majority of the latter patients had undiagnosed fibroids in the uterus.

*Postmenopausal patients*

Of the postmenopausal women, 282 were randomised to receive TAM and 306 to the control group. Figure 3 shows the curves for overall survival at 10 years for the two groups ($P$=0.73).

When survival curves are compared in the three subgroups by node status, there is a consistent trend in favour of TAM but never approaching statistical significance in any sub-group.

Table I shows an analysis done to assess the effect of TAM on the occurrence of events using the whole series of 961 evaluable patients. An event has been defined previously as the first evidence of relapse, whether it be local recurrence or distant metastases or death before a relapse is recorded. Local recurrence was only counted if it preceded distant metastases. In Table I it will be seen that patients treated with TAM fared substantially better and though the differ-

**Figure 3** Overall survival of 588 postmenopausal patients at 10 years.

**Table I** Log rank analysis of events in 961 patients by lymph node status

| *Node status* | *Group* | *Pts.* | *Obs.* | *Events exp.* | *O/E* |
|---|---|---|---|---|---|
| Negative | TAM | 146 | 35 | 40.14 | 0.87 |
| | Irrad. menop. / Control | 151 | 46 | 40.86 | 1.13 |
| Positive | TAM | 176 | 86 | 96.29 | 0.89 |
| | Irrad. menop. / Control | 173 | 99 | 88.71 | 1.12 |
| Not known | TAM | 159 | 60 | 61.64 | 0.97 |
| | Irrad. menop. / Control | 156 | 57 | 55.36 | 1.03 |
| ALL | TAM | 481 | 181 | 198.06 | 0.91 |
| | Irrad. menop. / Control | 480 | 202 | 184.94 | 1.09 |

$\chi^2=3.08$; 1 df; $P=0.07$.

ence was not statistically significant ($P=0.07$) it must be remembered that the 'controls' included the patients treated with an irradiation menopause.

### *Treatment at relapse*

It was difficult to insist on cross-over hormonal treatment for premenopausal patients as often these patients had rapidly progressing disease when they relapsed. Of those patients originally allocated to TAM, 47% had an irradiation menopause on relapse and 19% were represcribed TAM. Only 18% of the irradiation menopause group had TAM subsequently, the majority having chemotherapy. In the postmenopausal women, on the other hand, the disease at relapse was much more slowly progressive and 73% of the control patients were given TAM subsequently. Of the remainder, 25% were represcribed TAM; these were patients who did not relapse for at least 2 years following discontinuation of adjuvant TAM.

### *General side effects*

Both in pre- and post menopausal women, the side effects of TAM were minimal. A total of 2% of women required to reduce the dose to 10 mg daily and 4% stopped the drug in less than a year. None of the patients prescribed TAM on relapse required to modify the dose or stop the drug. The effects of TAM on menstruation have been noted above.

### *Second primary tumours*

Table II shows the incidence of second malignancies occurring in premenopausal women within the TAM and irradiation menopause groups. It can be seen that there is no significant difference in the incidence between the two groups. Table III show the incidence in the postmenopausal women and again there is no significant difference between TAM treated patients and controls. Furthermore there does not appear to be any specific increase in cancers of target endocrine organs in patients treated with TAM.

**Table II** Incidence of second primary tumours pre-menopausal

| | Allocated group | |
|---|---|---|
| *Type* | *TAM* | *Irrad.menop.* |
| Breast | 2 | 1 |
| Stomach | 1 | 0 |
| Thyroid | 1 | 0 |
| Cervix | 0 | 1 |
| Lymphoma | 0 | 1 |
| Total | 4/199=2% | 3/174=1.7% |

**Table III** Incidence of second primary tumours post menopausal

| | Allocated group | |
|---|---|---|
| *Type* | *TAM* | *Control* |
| Breast | 6 | 7 |
| Cervix | 1 | 2 |
| Endometrium | 1 | 1 |
| Rectum | 2 | 1 |
| Bladder | 1 | 2 |
| Skin | 1 | 1 |
| Stomach | 2 | 0 |
| Ovary | 1 | 0 |
| Colon | 1 | 0 |
| Thyroid | 0 | 1 |
| Total | 16/282=5.6% | 15/306=5% |

### Discussion

The present paper continues to show that patients treated with TAM have fewer events over the follow-up period compared to non-TAM treated patients. However at 10 years this difference is not statistically significant as it was at 7 years of follow-up. The TAM treated premenopausal patients may have had a significantly better disease free survival compared with a true control group given no adjuvant treatment, but it was considered unethical to have such a group when the trial was initiated.

At the inception of the trial, there was no reliable evidence to suggest how long TAM should have been prescribed on an adjuvant basis, so for the purposes of the trial a period of one year was chosen on an empirical basis. Since that time, evidence has been emerging that strongly suggests that TAM given for two years or more will make a significant difference to overall and disease free survival of treated patients.

Firstly, in the 6 year results of the NATO Trial of Adjuvant Tamoxifen (NATO 1985), it was shown that TAM given for two years, produced a highly significant reduction in mortality and an equally significant prolongation of the disease free interval in treated patients compared to controls ($P=0.0001$).

More recently, in a Scottish Breast Cancer Trial (MRC, 1987) it was shown that patients treated with TAM over a period of 5 years had a highly significant relapse free survival when compared to controls ($P=0.0001$).

Some laboratory work on the duration of TAM therapy has also been published (Jordan *et al.*, 1980). These workers compared a short course of TAM (50 $\mu$g daily for 4 weeks) with continuous therapy administered to rats with DMBA-induced mammary carcinoma. In the experiment, the majority of the rats given continuous therapy remained tumour-free unlike those on the short course. The workers felt that while there may be fundamental differences between the rat model and human breast carcinoma, the principles for the control of hormone-dependent growth may be similar.

Further clinical trials are now in progress comparing the administration of TAM for two years or more with TAM prescribed for life. The long term results of these trials will be awaited with interest.

Our thanks are due to the clinicians who participated in the trial, a list of whom has been previously published.

Thanks are also due to the Dept. of medical illustration and to Mrs P.A. Jones for secretarial assistance.

### References

JORDAN, CRAIG V., ALLEN, K.E. & DIX, C.G. (1980). Pharmacology of Tamoxifen in laboratory animals. *Cancer Treat. Rep.*, **64**, 745.

NOLVADEX ADJUVANT TRIAL ORGANISATION (1985). Controlled trial of Tamoxifen as single agent in management of early breast cancer; six year analysis. *Lancet*, **i**, 836.

RIBEIRO, G. & SWINDELL, R. (1985). The Christie Hospital Tamoxifen (Nolvadex) Adjuvant Trial for operable breast cancer – 7 year results. *Eur. J. Cancer Clin. Oncol.*, **21**, 897.

SCOTTISH BREAST CANCER TRIALS OFFICE (MRC) EDINBURGH (1987). Adjuvant Tamoxifen in the management of operable breast cancer. *Lancet*, **ii**, 171.